# Influence of different UV spectra and intensities on yield and quality of cannabis inflorescences

**DOI:** 10.3389/fpls.2024.1480876

**Published:** 2024-12-17

**Authors:** Daniel Stefan Huebner, Marat Batarshin, Sebastian Beck, Leon König, Inga Mewis, Christian Ulrichs

**Affiliations:** ^1^ Division Urban Plant Ecophysiology, Faculty of Life Sciences, Humboldt-Universität zu Berlin, Berlin, Germany; ^2^ Department of Chemistry, Humboldt-Universität zu Berlin, Berlin, Germany

**Keywords:** cannabis, UV radiation, secondary plant metabolites, yield, quality, cannabinoids, terpen

## Abstract

The raising economic importance of cannabis arouses interest in positively influencing the secondary plant constituents through external stimuli. One potential possibility to enhance the secondary metabolite profile is the use of UV light. In this study, the influence of spectral UV quality at different intensity levels on photomorphogenesis, growth, inflorescence yield, and secondary metabolite composition was investigated. Three UV spectra with five different intensities were considered: L1 (UVA:B = 67:33, 4.2 W/m^2^), L2 (UVA:B = 94:6, 4.99 W/m^2^), L3_1 (UVA:B = 99:1, 1.81 W/m^2^), L3_2 (UVA:B = 99:1, 4.12 W/m^2^) and L3_3 (UVA:B = 99:1, 8.36 W/m^2^). None of the investigated UV treatments altered the cannabinoid profile. Regarding the terpenes investigated, light variant L3_1 was able to positively influence the terpene profile. Especially linalool (+29%), limonene (+25%) and myrcene (+22%) showed an increase, compared to the control group without UV treatment. Growth and leaf morphology also showed significant changes compared to the control. While a high UVA share increased the leaf area, a higher UVB share led to a smaller leaf area. Of the UV sources examined, only L3_1 with 1.81 W/m^2^ and a radiation dose of 117.3 kJ m^2^ d^-1^ is suitable for practical use in commercial cannabis cultivation. The terpene concentration for this group was in part significantly increased with constant yield and cannabinoid concentration.

## 1 Introduction

*Cannabis sativa* is now legally cultivated in many countries. In addition to the use of the plant as a recreational stimulant, the medical use is increasingly in focus. Secondary metabolites of the plant are applied as active ingredients against various ailments. Here, the focus is so far on cannabinoids and terpenoids. Depending on the composition of these secondary metabolites, i.e. depending on the specific chemotype of the plant, the effect and the field of application may vary. For commercial cultivation, it would be advantageous to be able to directly influence the cannabinoid profile of the plant. One way to achieve this could be the use of short-wave radiation (Lydon et al., 1987).

Light not only serves as an energy source for photosynthesis in plants, but is also an essential environmental signal that activates a group of signal-transmitting photoreceptors that control plant growth, development, and metabolism (Karpiński et al., 2013). Plants respond to different light environments using different types of photoreceptors. These include phytochromes (PHYs) for red and far-red light, cryptochromes (CRYs) for blue and UVA light, phototropins (PHOTs), proteins of the slow-motion family and UVR8, a receptor for UV-B light (Galvão and Fankhauser, 2015). Once activated by light, photoreceptors interact closely with other photoreceptors or factors such as HY5, COP1 and PIFs, which play a crucial role in controlling plant shape and metabolism throughout their life cycle (Saijo et al., 2003). The use of UV-A and far-red light opens new ways to influence the shape and physiological functions of plants, which depends on the wavelength, intensity, and duration of irradiation (Fukuyama et al., 2017; He et al., 2021).

In previous studies, the use of short-wave radiation in the nm range between 100 nm and 400 nm (UV radiation) had an influence on flavonoids and phenolic acids in particular (Neugart and Schreiner, 2018; Agati and Tattini, 2010). Studies on cannabinoids showed mixed results so far. While older studies reported a linear influence of UV radiation on cannabinoid profiles (Caldwell, 1971; Lydon et al., 1987), more recent studies failed to demonstrate this relationship (Rodriguez-Morrison et al., 2021; Llewellyn et al., 2022; Westmoreland et al., 2023). In addition, use in commercial cannabis production is only of interest if quantities of yield do not decrease at the same rate as product quality increases. This would negate the gained advantage and not justify the invested effort.

Even if current studies have not yet found any commercially interesting fields of application for UV light in medical cannabis production, the variations of UV application are far from exhausted. Even if factors such as spectral quality, intensity, irradiation duration, irradiation time by day or cultivation section, etc. were investigated. However, the problem of comparability of these studies remains. The genetic variance has a considerable influence on the reaction of the metabolism to the environmental factors investigated, in this case UV radiation. In the following experiment, therefore, an unprecedented subdivision into different spectral qualities was made and examined for differences in the morphology and chemical composition of the plants.

While the above-mentioned studies (Rodriguez-Morrison et al., 2021; Westmoreland et al., 2023) have so far only considered different intensities of UV radiation or irradiation duration, but not the composition of UV light, the question arises whether different compositions of UV light also cause different physiological processes.

The objectives of the study were therefore:

*To investigate the influence of different UV spectra and intensity on morphology, inflorescence yield and secondary metabolite composition.*

## 2 Materials and methods

For this scientific study, three different experiments were used, to scientifically evaluate the effect of UV radiation on growth and quality parameters. While the majority of the test conditions were identical in all three trials, the following methods section will focus in particular on the differences between the different setups in order to ensure a detailed discussion of the results.

### 2.1 Experimental setup

All three experiments were carried out on the premises of the Humboldt University of Berlin. While experiments 1 and 2 were carried out simultaneously on one test area, experiment 3 was carried out in a separate climate chamber.

### 2.2 Plant material and environmental conditions

For all three experiments, cuttings from genetically identical mother plants of the EU-certified Cannabis sativa L. variety Fedora 17 were cut and placed under humidity domes and fluorescent lamps (LUMILUX T8 58W 840; OSRAM GmbH, Munich, Germany) at ~80 µmol m^2^ s^-1^ PPFD for 15 days for rooting. In experiments 1 and 2, 11 biological replicates (plants) were used per light treatment. In experiment 3, each light treatment group comprised 9 biological replicates. To avoid light interference between the treatment groups, all groups were spatially separated from each other by opaque foils. This arrangement ensures the independence of the treatments and minimizes possible interference effects. The Fedora 17 variety was selected due to its approval as an EU-certified variety and its genetic stability. This stability enables reproducible results, which are essential for scientific studies. In contrast to typical industrial hemp varieties, this phenotype was specifically selected for the production of CBD-rich flowers. Although Fedora 17 is considered monoecious, the phenotype used in this study does not develop male flowers, allowing for consistent production of female flowers. These characteristics make it particularly suitable for studying the effects of UV radiation on flower quality, terpen and cannabinoid profile. The rooted cuttings were then transferred to 15 L- square pots with a substrate of peat and perlite in the ratio 70% peat to 30% perlite. The plants were then divided equally and randomized between the different lighting groups. The temperature was 26 ± 2°C, measured for experiment 1 and 2 with a multisensor solution CERES (CERES, Hortiya UG, 10247, Berlin) and for experiment 3 with a HOBO datalogger (MX1104). The CO_2_ concentration was constant throughout the different studies (~400 ppm) also measured with CERES and HOBO datalogger. An oscillating fan with a diameter of 20 cm and a speed of 2,300 rpm (Monkey Fan Oscillating 20 W, secret jardin, AGOMOON Sprl, Audergheim, BEL) was placed in the upper corner of each lighting group of every experiment.

### 2.3 Illumination and UV treatments

In all experiments, different spectral UV compositions as well as control groups without UV treatment were tested, and the illumination and UV parameters are summarized in **Table 1**. Plants in all experiments were illuminated with a Photosynthetically Active Photon Flux Density (PPFD) of ~400 µmol m^2^ s-^1^ for 18 hours per day during the vegetative phase for 34 days. In the subsequent generative phase, which ran for 54 days, the illumination was reduced to 12 hours per day but increased to ~600 µmol m^2^ s-^1^ PPFD. The UV treatment started from the initiation of the generative phase (Exp. 1) or already with the vegetative phase (Exp. 2 and Exp. 3) and was applied over the entire lighting interval.

**Table 1:** Summary of the light and UV treatment parameters for experiments 1-3.

Treatment	Spectrum Composition (UVA: UVB)	Growth Phase	PPFD (µmol m^2^ s^-1^)	UV Intensity (W/m^2^)	Daily UV Dose (kJ m^-2^ d^-1^)	Daily UV Dose (mol m^-2^ d^-1^)
L1	67:33	Vegetative	400	–	–	–
		Generative	600	4.2	179.9	0.43
L1_C	Control	Vegetative	400	–	–	–
		Generative	600	–	–	–
L2	94:6	Vegetative	400	4.99	322.8	0.123
		Generative	600	4.99	215.2	0.082
L2_C	Control	Vegetative	400	–	–	–
		Generative	600	–	–	–
L3_1	99:1	Vegetative	400	1.81	117.3	0.006
		Generative	600	1.81	78.2	0.004
L3_2	99:1	Vegetative	400	4.12	266.9	0.014
		Generative	600	4.12	177.9	0.009
L3_3	99:1	Vegetative	400	8.36	541.7	0.028
		Generative	600	8.36	361.2	0.019
L3_C	Control	Vegetative	400	–	–	–
		Generative	600	–	–	–

UV radiation was provided by Lumitronix UV-LED chips and measured using a UVpad spectroradiometer (Opsytec Dr. Gröbel GmbH, Ettlingen, Germany). The specific UV intensities and daily UV doses for the respective spectral compositions and phases are shown in **Table 1**. In experiment 1, the ratio of UVA to UVB was 67:33 at a UV intensity of 4.2 W/m^2^, in experiment 2 94:6 with an intensity of 5.0 W/m^2^ and in experiment 3 for the variants L3_1, L3_2 and L3_3 99:1 at intensities of 1.8 W/m^2^, 4.12 W/m^2^ and 8.36 W/m^2^ respectively. The weighting factors according to Flint and Caldwell (2003) were used to calculate the biologically relevant UV photon flux (UVPFDBE).

### 2.4 Growth measurements and visual observations

The height of the individual plants (length of main stem from substrate surface to the highest point) was measured for experiment 3 within the groups L3_1, L3_2, L3_3, and the control group L3_C after 5 weeks at the end of the vegetative phase. This was repeated for all groups of all three experiments at the end of the generative phase. To determine the leaf area, after treatment week 4, leaf size was analyzed with ImageJ 1.42 software as described in (Rodriguez-Morrison et al., 2021). To determine the individual leaf size. The basis for the analysis were the youngest, fully developed fan leaves of the plants. The plants were visually inspected at least once a week for visible changes. These included morphological changes such as: leaf texture, upward curvature of leaf margins, leaf gloss, coloration of stigmas, trichome discoloration, leaf epinasty, pest infestation and disease symptoms and deficiency symptoms.

### 2.5 Harvest and yield measurements

After 88 days, all treatment plants were harvested by cutting off the stems at substrate level. The weight of the fresh flowers was determined gravimetrically for each test plant (PCD 10K0.1 balance, Kern & Sohn GmbH, Balingen-Frommern, Germany). The flowers were divided into main flower and lateral inflorescences. The weight was first determined on a fresh undried basis and then again dry.

For the analysis of phytocannabinoids and terpenes, 6 of the 9 test plants of each treatment group from experiment 3 were randomly selected.

In experiments 1 and 2, all test plants of the individual groups were analyzed for their cannabinoid content.

The flowers were cleaned of fan leaves, trichome-poor leaf tips and shoot pieces with scissors and transferred to appropriately labeled paper bags. Before storage at -80°C, all samples were shock-frozen by adding liquid nitrogen. The samples were subjected to three days of freeze-drying under vacuum (Alpha 2-4 LSCplus, LyoCube 4-8; Martin Christ GmbH, Osterode am Harz, Germany).

### 2.6 Flower quality

For the analyses of cannabinoids by high-performance liquid chromatography (HPLC) and for terpenes by gas chromatography (GC), freeze-dried flower material was transferred to sample vessels and pulverized with five stainless steel balls each in a vibrating mill (MM400; RETSCH GmbH, Haan, Germany). A frequency of 30 beats per second was selected, with one minute of vibration. The grinding process was repeated a second time for 30 seconds to ensure that the consistency of the plant material was as homogeneous as possible. The HPLC analysis was carried out using an Ultimate 3000 system. The chromatograms were analyzed and evaluated using Chromeleon 7.2 software (equipment and software provided by Thermo Fisher Scientific, Dreieich, Germany). The GC analyses was performed on an Agilent 7890B GC from Agilent Technologies, Inc (Santa Clara, CA, USA). The dedicated software of the instrument manufacturer Agilent Masshunter (version B.07.06.2704), for data acquisition control, and Agilent Masshunter Quantitative Analysis (version B.09.00), for quantification, was used.

For cannabinoid extraction, 20 mg of the powdered flower material (balance MC1 Analytic AC 120 S; Sartorius, Göttingen, Germany) was first transferred to 2 ml reaction vessels. Then 750 μl of a mixture of methanol and chloroform (9:1, v/v) was pipetted and vortexed for 3 seconds (ZX4; VELP Scientifica Srl, Usmate Velate MB, Italy). The vessels were then incubated for ten minutes in a thermal shaker (TS pro; CellMedia GmbH & Co. KG, Zeitz, Germany) at 500 rpm and a temperature of 20°C. Centrifugation (Heraeus Multifuge X1R with rotor FIBERLite F21-48 x 1.5/2.0, 21000 rpm = 48000 x g; Thermo Fisher Scientific, Waltham, USA) was then carried out for 5 minutes at room temperature (20°C) and 10,000 rpm. The supernatant was transferred to a new reaction vessel and the flower material, remaining as a pellet, was re-extracted twice (analogous to the process described, only with 500 µl instead of 750 μl of the methanol-chloroform mixture), whereby the extracts of one sample were pooled. This solution was then concentrated to near-dryness under a stream of nitrogen. The residues were dissolved in 500 μl acetonitrile and, after brief vortexing, centrifuged for 5 minutes with a Spin-X filter with a mesh size of 0.22 μm (LMS Consult GmbH & Co. KG, Brigachtal, Germany) and a rotation speed of 4,600 rpm and room temperature. HPLC analyses were performed at an oven temperature of 35°C. A 5 μm AcclaimTM 120 250-2.1 RP18 (Thermo Fisher Scientific) was used as separation column. A mixture of water and 0.85% formic acid (A) (v/v) or acetonitrile and 0.85% formic acid (B) (v/v) was selected as the eluent. The applied gradient was as follows: 70% B for 3 min, from 70% to 85% (B) in 7 min, from 85% to 95% in 7 min, 95% to 100% for 1 min, from 100% to 70% in 10 min. The detection of the selected phytocannabinoids cannabinolic acid (CBNa), cannabidiolic acid (CBDa) and Δ9-tetrahydrocannabinolic acid (D9THCa), such as the decarboxylated phytocannabinoids cannabinol (CBN), cannabidiol (CBD) Δ8-tetrahydrocannabinol (D8THC) and Δ9-tetrahydrocannabinol (D9THC) was carried out at wavelengths of 220 nm and 265 nm. External standards (Dr. Ehrenstorfer; LGC Standards, Augsburg, Germany) were used as a reference for quantification, from which a dilution series was prepared and equally analyzed by HPLC so that a dose-response curve could be generated. The response factor (RF) was calculated by the slope of the standard curve and used to determine the cannabinoid content.

For terpene extraction in experiment 3, about 100 mg of powdered flower material was transferred to 2 ml reaction vessels. A 500 μl solution of internal standard (ISTD) of carvacrol in isooctane (ratio 1:2,000; v/v) was pipetted onto this and vortexed for 5 seconds to mix the sample homogeneously with the solution. The samples were then incubated for 10 minutes in an ultrasonic bath (SONOREX, BANDELIN electronic GmbH & Co. KG, Berlin, Germany) on ice and subsequently centrifuged for 5 minutes at a temperature of 4°C and a rotation speed of 10,000 rpm. The supernatant was transferred to a new reaction vessel and the flower material remaining as a pellet was re-extracted twice (analogous to the process described, only with 250 µl instead of 500 μl of the ISTD mixture), whereby the supernatants were pooled. This solution was then concentrated to near-dryness under a stream of nitrogen. The residues were dissolved in 300 μl isooctane for subsequent GC analyses. Samples were stored in a freezer until GC analysis. Gas chromatography was performed using an Agilent DB5-MS separation column from Agilent Technologies (30 m; 0.32 mm ID; film thickness: 0.25 μm). Detection was performed using a flame ionization detector (FID) at 280°C, while the injector temperature was set to 250°C. A sample volume of 1 μl was injected in a 1:20 split. Helium was used as the carrier gas at a constant flow rate of 2 ml/min. The selected temperature program was as follows: 45°C for 5 min, 8°C/min to 200°C and then 200°C for 10 min. Quantification was performed using carvacrol as an ISTD individual calibration curves of chemical standards for the monoterpenes β-myrcene, limonene, linalool and α-pinene as well as the sesquiterpene ß-caryophyllene in between 1 and 400 ng/µl).

### 2.7 Statistical analysis

The statistical analysis was performed with RStudio (RStudio Team (2020). RStudio: Integrated Development for R (RStudio, PBC, Boston, USA). Necessary packages for statistical analysis were installed, using standard packages as well as specialized ones such as dplyr, ggplot2, car and multcomp. Data were tested for normal distribution of the dependent variable within each group using visual methods and the Shapiro-Wilk test. Homogeneity of variances was tested using Levene test. Depending on whether normal distribution and variance homogeneity were fulfilled, a one-factorial ANOVA or a Student’s T-test was performed. If the assumptions of normal distribution or variance homogeneity were not met, the Kruskal-Wallis test or the Wilcox test was used. Significant results were further investigated by *post-hoc* analyses, with the TukeyHSD and Dunett test, to determine which groups differed. The results yielded F and p values, with a significant p value indicating significant group differences (p value < 0.05).

## 3 Results

### 3.1 Morphology

The results of the observations and measurements carried out are presented in the following results section. Starting with the morphological changes.

#### 3.1.1 Height growth

Continuous observation with the CERES sensors made it possible to document and evaluate daily changes in morphology in experiments 1 and 2.

**Figure 1:**
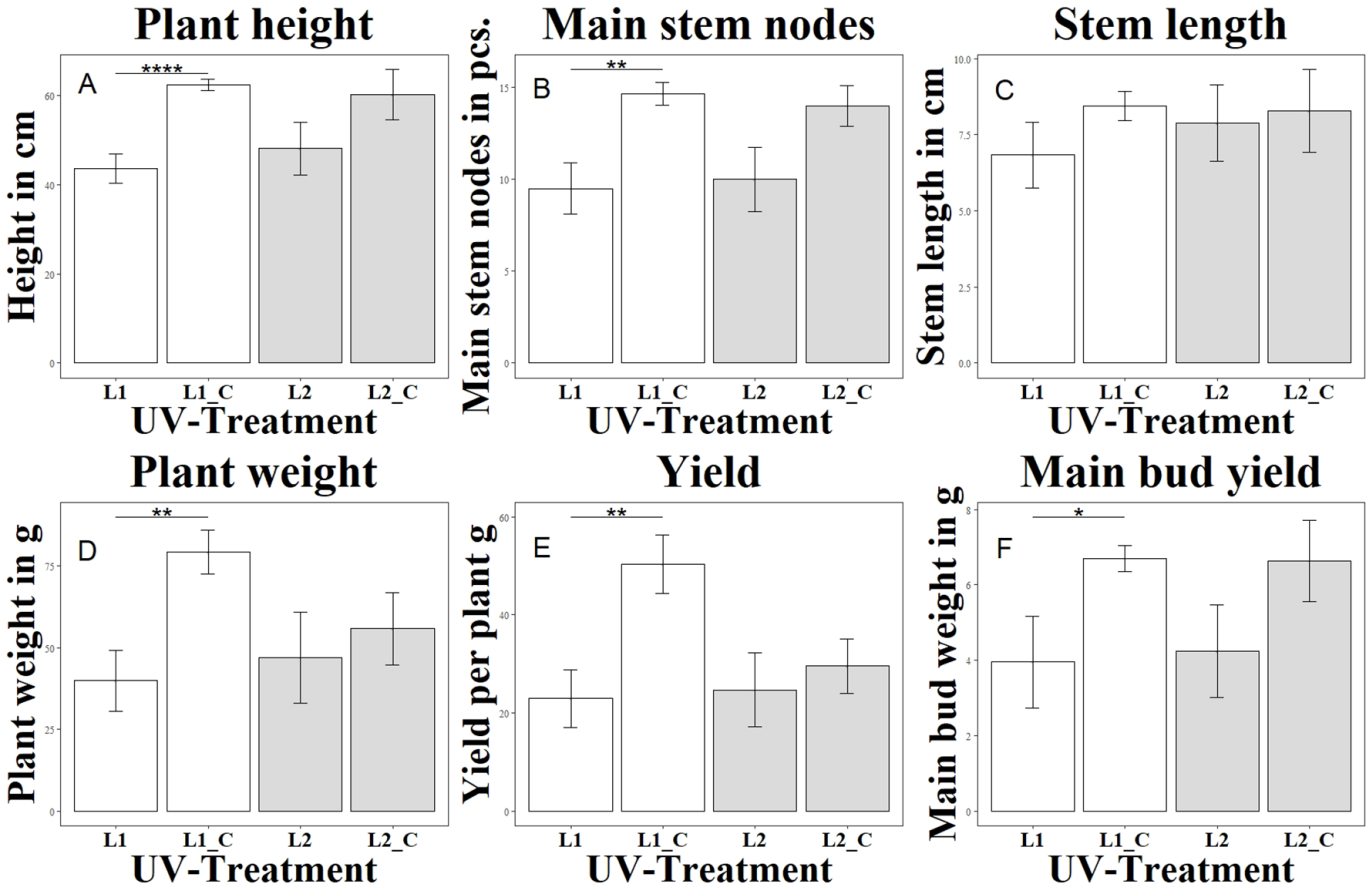
Changes in morphological and yield parameters as a function of UV exposure. Each letter **(A–F)** represents a different parameter: **(A)** shows plant height (Height in cm), **(B)** main stem nodes (in pcs.), **(C)** stem length (in cm), **(D)** plant weight (in g), **(E)** total yield per plant (in g), and **(F)** main bud yield (in g). Different UV treatments were applied: L1 with a UVA:UVB ratio of 67:33 (4.2 W/m^2^), L1_C: Control without UV, L2 with a high UVA:UVB ratio of 94:6 (4.99 W/m^2^), L2_C: Control without UV. The bar graphs indicate significant differences in these parameters under varying UV conditions. Significance levels: **** (P ≤ 0.0001), ** (P ≤ 0.01), * (P ≤ 0.05).

**Figure 2:**
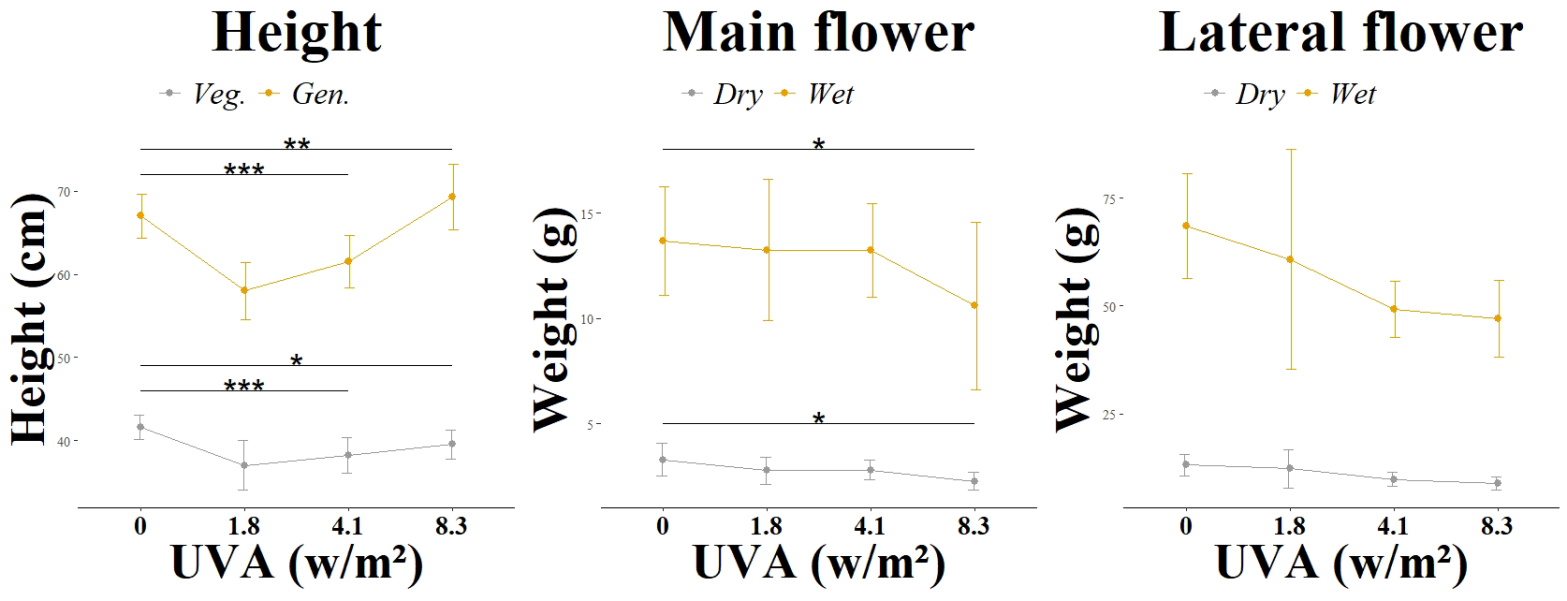
Morphological changes according to light group. Different UV intensities and ratios were tested: L3 main flower and L3 lateral flower in a UVA: UVB ratio of 99:1 and at varying intensities (0.0 W/m^2^, 1.81 W/m^2^, 4.12 W/m^2^, 8.36 W/m^2^). Significance level: *:(P ≤ 0.05), **: (P ≤ 0.01), ***: (P ≤ 0.001).

The group (L1) differed significantly from the variant without UV (L1_C) in terms of height growth on the day of harvest, as shown in **Figure 1**. Already from week 2 of the UV treatment with a UV-AB ratio of 67:33, a growth stagnation was observed here compared to the untreated control (L1_C). The elongation growth at the beginning of the generative phase was less pronounced than in the control group (L1_C). This trend continued until the end of the cultivation period. In contrast to the group (L2) with a UV-AB ratio of 94:6, which showed no significant differences in height growth compared to the control variant without UV (L2_C). In experiment 3, the morphological changes were checked and documented manually every second day. Compared to the control group without UV treatment (L3_C), the growth of (L3_1) and (L3_2) was significantly reduced, as shown in **Figure 2**. Stagnation of growth was already observed after the end of the vegetative phase. While the difference to the control was highly significant in group (L3_1), the difference in group (L3_2) was only significant. The observed stagnation persisted even after the end of the generative phase. Group L3_3 did not differ significantly from the control group L3_C. Overall, a growth depression was initially observed in the plants treated with UVA radiation. However, this growth reduction decreased with increasing UVA radiation and after completion of the generative phase in the L3_3 group even exceeded the mean growth height of the untreated plants (L3_C).

#### 3.1.2 Leaf morphology

Different effects of UV treatment were also observed on leaf morphology. Similar to height growth, differences in the spectral composition of UV radiation led to different physiological reactions.

During the first three days of UV treatment in experiment 1 with a daily biologically active UV dose of 0.43 mol m^2^ d-^1^, the leaves of the plant in group L1 curled. From the fourth day onwards, irreversible damage to the leaf tissue was observed, initial chlorotic spots die off and become necrotic. A considerable part of the leaves is damaged, new shoots show little to none of this damage. Although bioactive UV radiation remains high in the L1 group, leaves and shoots growing from the second week of treatment are not affected by necrotic spots or leaf death. In addition, epicuticular wax was observed on the leaves from the second week after UV treatment. Especially newly grown leaves showed this effect. In contrast, no leaf curling and atypical leaf damage such as chlorosis or necrosis could be observed in the control group (L1_C). The leaf area of the youngest, fully developed fan leaves of the group (L1) was reduced by 36.6% compared to the control group (L1_C) after treatment week 4.

In contrast, group L2 in experiment 2 with the lower UVB level and a daily biologically active UV dose of 0.123 mol m^2^ d-^1^ showed no necrotic changes in the vegetative phase. However, the leaf size was reduced by about 16.6% in the UV variant. In addition, leaf curling was observed from the beginning of the second week of UV treatment but was less visible compared to the L1 group. In addition, the tendency to curl was less pronounced in younger leaves over the course of the trial. Also, in experiment 3 with the continuously increased daily biologically active UV dose (0.006, 0.014 and 0.028 mol m^2^ d-^1^) the plants treated with UV light showed leaf morphological differences compared to the control (L3_C).

One week after UV treatment, a layer of epicuticular wax was observed in the L3 group, but it was less pronounced than in the treatment group (L1). The leaf area increased with increasing UV intensity (-1.6%, +10.7%, +19.6%), in contrast to experiments 1 and 2. Neither chlorotic nor necrotic changes were observed in this experiment regardless of radiation intensity.

#### 3.1.3 Flower formation, degree of ripeness and harvest weight

An effect of UV radiation was also observed in the formation of flowers in the first and second experiments. While in the group (L1) with a daily biologically active UV dose of 0.43 mol m^2^ d-^1^ in the generative phase, the first flower buds were already visible in the second week of generative exposure, in the control group (L1_C) it took 3-4 days longer until the first flower buds were visible. This effect was not observed in the group (L2) with a daily biologically active UV dose of 0.082 mol m^2^ d-^1^ in the generative phase. The flowers formed at the same time as in the control group (L2_C). At the time of harvest, the plants in the group (L1) had less brownish colored pistils than in the control variant (L1_C). This effect was also observed in the L2 group. However, the trichomes in both UV groups showed a greater number of brownish and thus senescent trichomes. While the UV spectrum of the group (L2) had no detectable negative effect on the total flower weight and the weight of the main flower, the L1 group showed a negative significant effect for both investigated attributes. The reduction in harvest volume was not statistically significant for either total weight or main flower weight in experiment 2. The influence of UVA radiation with a daily biologically active UV dose of 0.004 - 0.019 mol m^2^ d-^1^ in the generative phase in experiment 3 on flower formation was less pronounced than the UVAB effects already described. No time delay in flower induction was observed. Compared to the control group (L3_C), the weight of the main flower of (L3_3) was significantly reduced both dry and wet. The UV levels (L3_2) and (L3_1) had no significant effect on the weight of the main flower.

### 3.2 Secondary metabolites

#### 3.2.1 Cannabinoids

**Figure 3:**
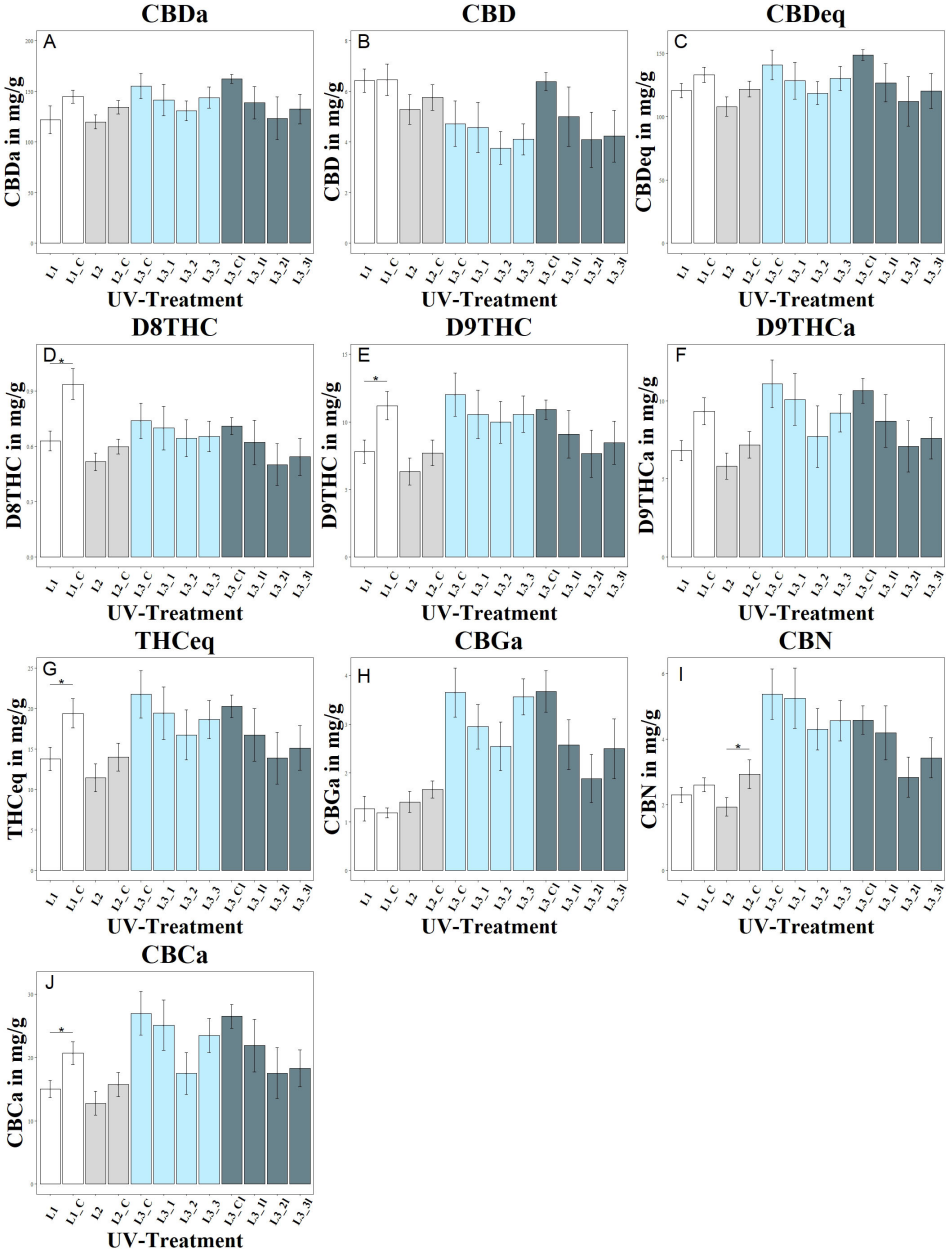
Changes in cannabinoid composition as a function of UV exposure. Each letter **(A–J)** represents a different cannabinoid: **(A)** shows CBDA, **(B)** CBD, **(C)** CBDeq (CBD + CBDA), **(D)** D8THC, **(E)** D9THC, **(F)** D9THCa, **(G)** THCeq, **(H)** CBGA, **(I)** CBN, and **(J)** CBCA. Different UV intensities and ratios were tested: L1 with a UVA:UVB ratio of 67:33 (4.2 W/m^2^), L1_C: Control without UV, L2 with a high UVA:UVB ratio of 94:6 (4.99 W/m^2^), L2_C: Control without UV, L3 in increasing UVA:UVB ratio (99:1) at varying intensities (L3_1: 1.81 W/m^2^, L3_2: 4.12 W/m^2^, L3_3: 8.36 W/m^2^), L3_C: Control without UV, L3l in increasing UVA:UVB ratio (99:1) at varying intensities but harvested lateral flowers (L3_1l: 1.81 W/m^2^, L3_2l: 4.12 W/m^2^, L3_3l: 8.36 W/m^2^), L3_Cl: Control without UV. The bar graphs indicate cannabinoid concentrations under these conditions. Significance level: * (P ≤ 0.05).

The study showed a significant negative effect of UV treatment in experiment 1 on the tetrahydrocannabinoids D8THC, D9THC and total THC in the L1 group, as shown in **Figure 3**. In addition, UV treatment had a significant negative effect on the CBCa concentration in group L1.

Trends for negative effects of UV irradiation were also observed within the second experiment, but these were less pronounced than in experiment 1. There was a tendency for lower CBD and CBDa levels in group L2 compared to the control group L2_C, although the levels of CBD, CBDa and CBDtotal were not significantly affected by UV treatment. UV irradiation had a significant negative effect on the CBN concentration in group L2.

The different UV intensities of the L3 groups in experiment 3 showed a decreasing trend in CBD and CBDa contents with increasing UV intensity, but without statistically significant differences between the intensities. UV irradiation showed no significant effect on the CBGa concentration but had a significant negative effect on the CBN concentration of the L2 group and the CBCa concentration in the L1 group. In the L3 groups, no significant effect of stepped UV irradiation on the CBN concentration was observed, although lateral CBN levels were lower in all L3 groups than in the corresponding main shoots.

#### 3.2.2 Terpenes

**Figure 4:**
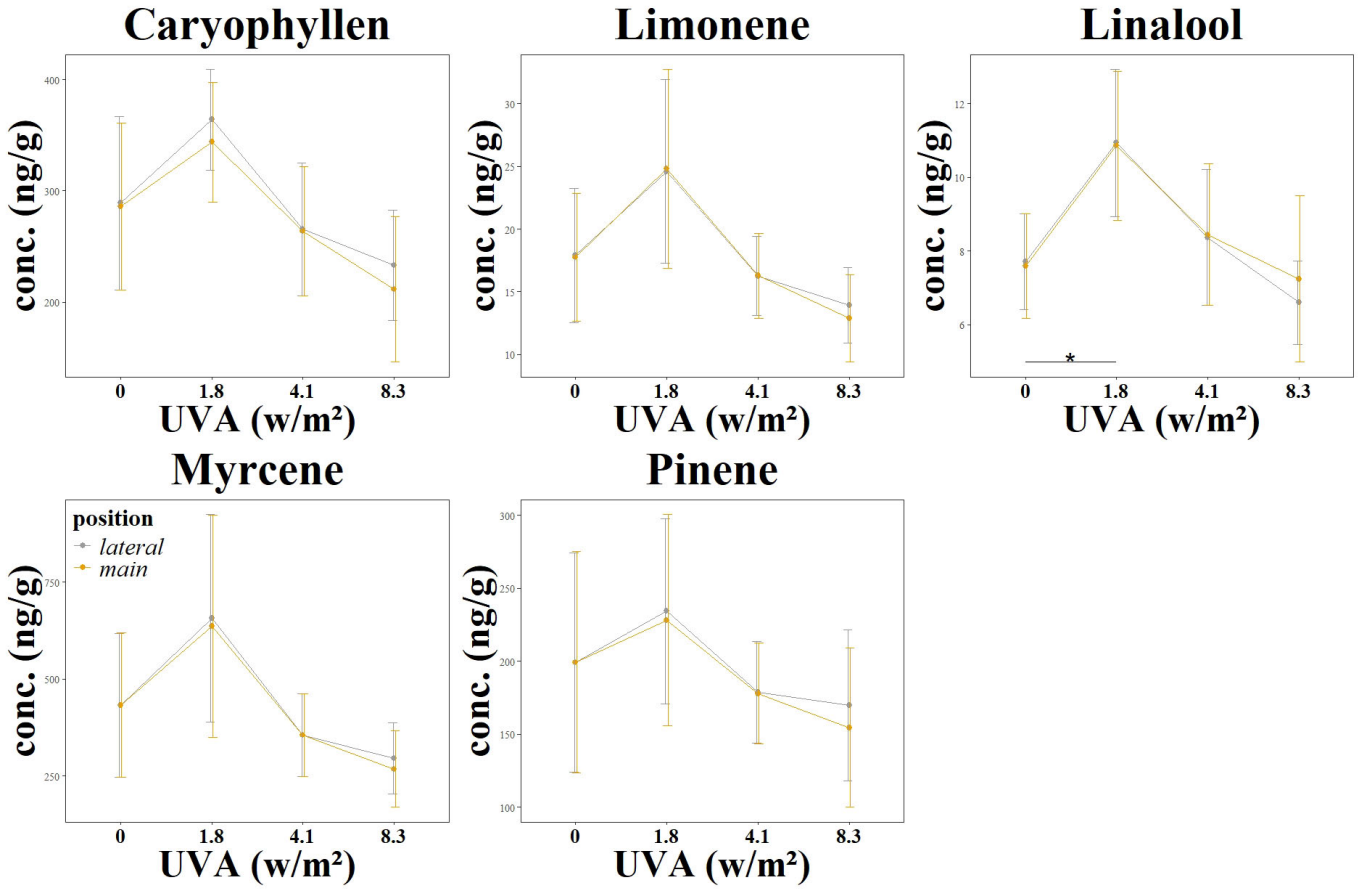
Changes in terpen concentrations as a function of UV exposure. Different UV intensities and ratios were tested: L3 main flower and L3 lateral flower using a UVA: UVB ratio of 99:1 at varying intensities (0.0 W/m^2^, 1.81 W/m^2^, 4.12 W/m^2^, 8.36 W/m^2^). The graphs show the resulting cannabinoid concentration changes. Significance level: *:(P ≤ 0.05) (Linalool 0) – (Linalool 1.8)*.

In addition to an analysis of cannabinoid content, the influence of continuously increasing UVA radiation on the terpene concentration was analyzed in the third experiment. The terpenes carophylene, limonene, linalool, mycrene and pinene were investigated, as shown in **Table 2** and **Figure 4**. While the change in concentration of all the terpenes mentioned showed a positive trend at an irradiance of 1.8 w/m^2^, this positive trend was only significant for linalool. This trend and also the significant change in linalool was observed both in the main flower and in the lateral flowers. However, the two treatment groups with medium (4.1 w/m^2^) and high (8.3 w/m^2^) UV radiation intensity showed no positive trend and did not differ significantly from the control group L3_C.

**Table 2:** Terpen concentrations (mg/g) by light group.

Treatment	Caryophyllene	Limonene	Linalool	Myrcene	Pinene
L3_C (Control)	285 ± 33	17 ± 2	7 ± 0.6	432 ± 83	199 ± 33
L3_1	343 ± 24	24.79 ± 3.5	10.85 ± 0.9	636 ± 128	228 ± 32
L3_2	263 ± 23	16.28 ± 1.4	8.44 ± 0.8	355 ± 43	177 ± 14
L3_3	211 ± 29	12.87 ± 1.6	7.24 ± 1	267 ± 44	154 ± 24
L3_Cl (Control/lateral)	288 ± 34	17.90 ± 2.4	7.70 ± 0.5	431 ± 83	199 ± 33
L3_1l (lateral)	364 ± 20	24.59 ± 3.3	10.93 ± 0.8	656 ± 120	234 ± 28
L3_2l (lateral)	265 ± 24	16.26 ± 1.3	8.36 ± 0.7	355 ± 43	178 ± 14
L3_3l (lateral)	233 ± 22	13.91 ± 1.3	6.60 ± 0.5	295 ± 40	169 ± 23

Given is the plant flower terpene content (dry weight) on the basis of GC analyses as a function of UV exposure. “Lateral” in this context refers to flowers growing on side branches rather than the main stem. Different UV intensities and ratios were tested: L3 main flower and L3 lateral flower in increasing UVA: UVB ratio of 99:1 and at varying intensities (0 W/m^2^, 1.81 W/m^2^, 4.12 W/m^2^, 8.36 W/m^2^).

## 4 Discussion

### 4.1 Applicability of biological effective weighting factors according to Caldwell

Current studies investigating the effect of UV treatment on cannabis plants apply biological spectral weighting factors to better quantify the disproportionate effect on morphology, physiology, and metabolism (Rodriguez-Morrison et al., 2021; Westmoreland et al., 2023). The applied weighting factors are based on a publication by Flint and Caldwell from 2003. The research by Flint and Caldwell on biological spectral weighting functions (BSWF) primarily focused on the effects of UV radiation on plant growth rather than on secondary plant metabolites. The applicability of the BSWF to the cannabis plant and its secondary metabolites, such as THC and CBD or various terpenes, has not been conclusively clarified. In the experiment, UVA treatment groups with a very small weighted UVPFD also showed significant effects on various parameter. To facilitate the interpretation of the results, **Table 3** shows the UV light applied in all common units. Both weighted and unweighted.

**Table 3:** Overview of the different light regimes used in this study.

Group UVA: UVB	Control (0:0)	L1 (67:33)	L2 (94:6)	L3_1 (99:1)	L3_2 (99:1)	L3_3 (99:1)
UV W m^-2^	0	4.2	4.99	1.81	4.12	8.36
UVA W m^-2^	0	2.82	4.64	1.80	4.10	8.30
UVB W m^-2^	0	1.38	0.35	0.01	0.02	0.06
UVPFD mmol m^-2^ s^-1^	0	12.45	15.15	5.19	11.83	24.0
UVPFDBE mol m^-2^ d^-1^	0	0.43	0.082	0.004	0.009	0.019
kJ m^-2^ d^-1^	0	179.9	215.2	78.2	177.9	361.2

The table shows the applied UV-radiation in different units.

### 4.2 Influence on growth morphology

UV light triggers a reaction in plants. However, the effects of spectral quality, intensity, timing, and duration on plant responses are largely unclear. UV intensities and plant growth in the L3 group imply a threshold value. If this value is exceeded, a counter-reaction is triggered in the plant, which compensates for the growth inhibited by UVA light. While the growth depression is significant with L3_2 and L3_1, the level of the control group L3_C can be reached with a UVA value of 8.3 W/m^2^ (L3_3), and even exceeded in the mean value. This effect is already apparent during vegetative growth and increases as the generative phase progresses until harvest. Other studies (Kang et al., 2022; Hu et al., 2022) have already shown that the intensity of UV light is a relevant influencing factor with basil and Chinese cabbage. The studies demonstrate an optimal limit range of UV radiation that triggers the desired response Judging by the data, this ideal range also exists for cannabis. While at 1.8 W/m^2^ (L3_1) and 4.1 W/m^2^ (L3_2) the amount of energy is too low to trigger a counter-reaction, 8.3 W/m^2^ (L3_3) is sufficient to trigger a mechanism in the plant that compensates for the contrary growth-reducing effects of UVA light. While UVB decreases net photosynthesis, recent studies (Neugart and Schreiner, 2018) show that UVA radiation can have a positive but also negative effect on net photosynthesis depending on the crop and light composition. The compensation of growth at group 8.3 w/m^2^ (L3_3) could be related to an increased photosynthetic rate, which is due to increased CO_2_ absorption through increased stomatal openings of the leaf (Sarlikioti et al., 2011). This leads to the growth of adaxial epidermal cells and to the observed increase in leaf area. The radiation doses of group L3_1 and L3_2 are not sufficient to have this effect on the leaves, but still lead to oxidative stress. This causes a reduction in quantum efficiency that cannot be compensated by an increased formation of epidermal cells. Similar observations have already been made in experiments with Chinese cabbage (He et al., 2021). The study describes that UVA photoreceptors, known as cryptochromes, have an essential function in the photosynthetic development of leaves. This occurs through the regulation of gene expression in chloroplasts. These genes are involved in the transcription and expression of other genes coding for photosystem II (PSII) (Petroutsos et al., 2016; Walters, 2005). However, excessive exposure to UVA radiation can potentially lead to impairment of the PSII protein complex and result in a reduction in quantum efficiency, which parallels the known effects of UVB radiation (He et al., 2021; Jansen et al., 1998). The experiment carried out follows this explanation, so the excessive UV radiation in group L1 led to a significant growth depression and reduced biomass weight.

The difference of the influence between moderate and excessive irradiation with UVB on morphology is evident in the comparison with the group L2 (UVA 93: UVB 7).

While the UV treatment of group L1 resulted in lower yields, smaller plants and physical damage at the cellular level compared to group L1_C, there were no significant differences at the morphological level in group L2 compared to the group that was not treated with UV radiation (L2_C). This was despite the higher UV intensity. Thus, the plant response is not directly related to the irradiance energy but is specifically dependent on the corresponding light quality.

### 4.3 Influence on leaf morphology

The influence of UV light on the morphology of the leaves was observed particularly in the L1 group. While the first three days the leaves of the plant curl up. Leaf curling under UVB radiation has already been observed in the model plant *Arabdopsis thaliana* (Fierro et al., 2015) and a recent publication investigating cannabis (Rodriguez-Morrison et al., 2021).

From the fourth day on, irreparable damage occurs. A large part of the leaves perishes, only the new shoots remain intact. The senescence of the leaves is due to a variety of effects that act on the plant when exposed to UVB light.

While UVB light is often associated with the formation of covalently linked pyrimidine residues in DNA, leading to cell death Britt, 1995; Shi and Liu, 2021). Additionally, the formation of reactive oxygen species (ROS) by UV light leads to oxidative stress through the oxidation of lipids and proteins. With long-term and excessive UVB radiation, this process can also lead to cell death. Low doses of UVB are associated with eustress, while increasing intensity increases the risk of cellular damage by ROS (Hideg et al., 2013). A decrease in photosynthesis can also be initiated by UV-B radiation. With increasing radiation, the maximum quantum yield of photosystem II continuously lowering (Shi and Liu, 2021; Sztatelman et al., 2015).

Although the L1 leaves grown under the influence of UV light had a smaller leaf area compared to the control group L1_C, the curling of the leaf edges was less pronounced and necrotic damage no longer occurred in the further course of growth.

The smaller leaf area caused by UVB radiation has already been observed in previous studies on cannabis (Rodriguez-Morrison et al., 2021). The impact of UVB on leaf morphology is partly due to its ability to induce stress responses in plants, leading to stunted growth and altered developmental pathways. This includes modifications in cell division and expansion, as well as changes in the production of protective compounds like flavonoids, which can alter leaf structure and function.

UVA had the opposite effect in the experiment. In the L3 groups, the leaf area index increased with increasing UVA intensity.

There are hardly any comparative studies with cannabis and UVA treatment. However, if the field of plants considered is extended, there is also a range of evidence for this morphological change in leaf area.

Studies have shown that UVA light can increase leaf area and leaf number in plants like spinach, cucumber, Chinese kale and tomato. This is often attributed to UVA’s role in photomorphogenesis, the light-mediated development process in plants (He et al., 2021).

For instance, exposure to UVA light has been shown to increase total leaf area and leaf-area index in plants like *Mentha piperita* (Maffei et al., 1999).

### 4.4 Adaptation of UV light

Despite the disproportionate amount of UVB in group L1, the weighted UVPFD of group L1 (0.4 mol m^2^ d-^1^) is about four times higher than on a summer day on 41° latitude and 1450 m elevation (0.1 mol m^2^ d-^1^) (Westmoreland et al., 2023), the plant has still been able to protect the young shoots of the plant. While the UVC radiation of the sun is almost completely filtered by the ozone in the earth’s atmosphere, UVB radiation is omnipresent in sunlight (Sztatelman et al., 2015). During evolution, some physiological strategies of the plant established themselves to deal with the potentially cell-damaging radiation. Starting with the genes *uvr*1, *uvr*2, *uvr*3 and *uvh*1. These genes are all involved in the DNA damage repair pathway and their produced compounds protect the plant from UVB mutations that could have negative effects for reproduction. In this context, *uvr*1 and *uvr*2 are involved in the repair of pyrimidine-pyrimidinone dimers but occur especially in the late flowering phase to ensure low mutation rates in male and female cell lineages. Another strategy is the formation of photoprotective secondary metabolites. Especially the formation of flavonoids is linked in the literature with the exposure to UV light. Flavanol, anthocyanins and proanthocyanidins are the most important representatives here (Rozema et al., 1997). To elicit a response to UVB radiation, a UVB receptor is required. This receptor was found with UVR8 and is significantly involved in UVB photomorphogenesis and UVB stress tolerance. UVR8 responds most strongly to wavelength 285nm and ultimately triggers the induction of ELONGATED HYPOCOTYL 5 (HY5) (Heijde and Ulm, 2012; Wilson and Greenberg, 1993). The response of the L1 group shows that a sudden onset of UVB radiation places the plant under acute stress and an initial response is initiated after only a few hours. The observed curling of the leaf fingers is attributed to an uneven inhibition of growth (Fierro et al., 2015). Leaves that are formed only during the UV irradiation phase are viable and epinasty of the leaf margins also no longer occurs. Instead, these leaves are significantly reduced in surface area, an effect directly related to UVR8 receptor (Fierro et al., 2015; Wargent et al., 2009).

### 4.5 UV influence on florescence morphology

The negative influence of UVB radiation can be observed here especially in the harvested flower weight. The yield of the L1 group with the greater UVB proportion is significantly reduced compared to the non-irradiated group. Since 90% of the plant’s dry mass is due to photosynthetic CO_2_ assimilation, flower formation is reduced due to the impairment of the PSII protein complex and a subsequent reduction in quantum efficiency (Chandra et al., 2008; Zelitch, 1975).

When looking at the L3 groups with the continuously increased UV share, there is a negative influence of UVA of the highest dosed UV group (L3_3) compared to the control (L3_C) on the flowering quantity of the main shoot. Both the fresh flowers and the dry quantity are affected. The lower side shoots are not affected by the UV effect and do not differ significantly from the control group in terms of quantity. This difference to the main shoot is probably due to the filtering properties of leaves, which reduce the measured UV quantity by around 20%. In addition, the greater distance from the UV lamp of the lower shoots automatically leads to a lower UV concentration.

In addition to the promotion of secondary constituents, influencing the time of harvest is also a possible application of targeted UV application. Both the L2 and L3_3 group produced the first signs of inflorescences early (3 days) after the start of photoperiodic flower induction. Group L1 and the lower dosed L3 groups did not show this effect. While these results have not yet been observed in cannabis, evidence of the effect of UV light on inflorescence formation is already known in the wider plant world (Reddy et al., 2004; Sampson and Cane, 1999). This effect is due to the photoperiodic signaling pathway, which mediates the perception of blue light by CRY1 and CRY2 on the one hand and by the blue light receptor FKF1 on the other (Valverde et al., 2004). CRY2 promotes flowering by promoting the stabilization of the transcriptional regulator CONSTANS (CO) (Mouradov et al., 2002). UVB is sensed by UVR8 and acts as an input signal to the circadian clock, which is involved in the photoperiod pathway (Fehér et al., 2011). However, these signaling pathways and receptors are only partially influenced by radiation. In addition to light quality, flower formation is subject to the control of other internal (autonomous ageing, gibberellins) and external signals (ambient temperature, vernalization and day length) (Huché-Thélier et al., 2016).

In addition to the influence on initial flower formation, a difference was also observed between the UV groups regarding the maturation of the flowers. An indicator for the degree of maturity of the cannabis flower is usually the condition of the trichomes, especially the glandular hairs with the heads. These are the main sites of cannabinoid production, and secondary metabolite content and appearance changes over time. Immature trichomes are clear and colorless. During flower development, trichomes increase in size and density. The optimum harvest time is reached when the majority of the trichomes have a cloudy, milky discoloration, while a few may still be clear or already have a brownish discoloration. If the trichomes are predominantly brownish in color, the flower is overripe (Tanney et al., 2021; Livingston et al., 2021). While the pistils of both L1 and L2 were less brown than the control group without UV treatment (L1_C and L2_C), a large number of the trichomes of the UV groups L1 and L2 were already brownish in contrast to the control.

Cannabinoids, when exposed to light, particularly UV light, can undergo various chemical changes, including degradation. The results indicate that this mechanism also plays a role during cultivation in the case of excessive treatment with UVB light. The increased turbidity of the trichomes was not observed in plants treated with UVA light (L3). Light therefore leads to a degradation of THCa to CBN. Since the CBN values were not increased in the experiment carried out, it is reasonable to conclude that alternative degradation pathways still exist.

### 4.6 Secondary metabolites

The effect of UV radiation on secondary metabolites has been scientifically proven (Neugart and Schreiner, 2018). Flavonoids and phenolic acids have photoprotective properties. They serve as both UV-absorbing shields and antioxidants (Agati and Tattini, 2010) and are thus able to counteract oxidative stress caused by UV exposure. Less clear is the role of cannabinoids in the defense response of short-wave radiation.

The experiments carried out show that UV radiation has a significant influence on the cannabinoid profile. In particular, the concentrations of THC showed a significant decrease when treated with UV radiation. Other cannabinoids, such as CBCa and CBN, also showed this negative trend. This change in the cannabinoid profile contradicts the actual goal of increasing the cannabinoid profile. In order to understand the underlying mechanisms, an understanding of cannabinoid synthesis is required.

The cannabis plant is capable of synthesizing over 180 different structures from the cannabinoid class (Hanuš et al., 2016). The biosynthesis of cannabinoids in the cannabis plant is a complex process in which a precursor compound is converted into several cannabinoid compounds. Most cannabinoids are synthesized from a compound called cannabigerolic acid (CBGa), which plays a central role in the formation of THCa and CBDa.

The starting material for cannabinoid synthesis is dimethylallyl pyrophosphate (DMAPP) and isopentenyl pyrophosphate (IPP). Enzymatic condensation from the two precursors geranyl pyrophosphate and olivetolic acid, catalyzed by an aromatic prenyltransferase (APT), forms cannabigerolic acid (Tahir et al., 2021). CBGa can be converted to other cannabinoid acids, such as tetrahydrocannabinolic acid (THCa) and cannabidiolic acid (CBDa), through various enzymatic reactions. THC, CBD, and CBC are present in the plant mainly in their acidic precursors tetrahydrocannabinolic acid (THCa), cannabidiolic acid (CBDa), and cannabichromenic acid (CBCa) (Tahir et al., 2021). These compounds are the precursors of THC and CBD and CBC. CBD and the acidic form CBDa, respectively, accounted for the largest proportion in the experiment conducted. In the following, the individual secondary components of the cannabis flower will be examined in more detail.

### 4.7 CBD

No influence of UV radiation on the CBD or CBDa concentration was observed for either the L1 group or the L2 group. However, the trend points more toward a negative influence than the desired improvement. The different intensities of the L3 group also had no significant influence on the cannabinoid CBD and its acidic precursor.

This is also consistent with the current state of studies (Rodriguez-Morrison et al., 2021; Llewellyn et al., 2022). Both studies could not quantify a positive effect of UV radiation on the CBD concentration of the plant. The ratio of CBD to CBDa was greater in the L1 group compared to the non-irradiated group L1_C (1:20.6 > 1:22.3). In contrast, the ratio of the L2 group was smaller compared with the unirradiated group L2_C (1:22.1 < 1:22.9). For group L3, the observation is reversed: as the intensity increases, the ratio of CBD to CBDa decreases. The comparison of the UV groups gives an indication that UVB radiation might contribute more to decarboxylation than UVA. Previous studies have shown that UV light, especially UVB and UVC, can have an accelerating effect on the decarboxylation of non-cannabinoids. This is attributed to the high-energy UV photons providing sufficient energy to cleave the weak bond of the carboxyl group (Filer 2022; Ge et al., 2010; Li et al., 2011; Pinna and Pusino 2012; Vaida et al., 2008; Wang et al., 2017; Zhang et al., 2018). The results of the experiment provide evidence that this principle may also be applicable to cannabinoids and is consistent with the observation of prematurely senescent trichomes.

### 4.8 THC

The THC concentration, which is composed of D9THC, D8THC and D9THCa, also did not differ significantly between L2 and control L2_C and L3 groups and control L3_C. Instead of an improvement, the UV treatment at L1 even led to a significantly reduction of D9THC, D8THC and THCtotal compared to the control L1_C. It is important to emphasize here that the genetics used are a commercial hemp variety. This variety is through breeding intervention not or only very limited capable of producing THC. The levels used for comparison are accordingly low. But also, here the result corresponds to current studies (Rodriguez-Morrison et al., 2021; Llewellyn et al., 2022; Danziger and Bernstein, 2021). Changes in cannabinoid profiles as a function of environmental factors, runs over several generations rather than one growing cycle. Thus, its use would not be suitable as a short-term means of influencing cannabinoid profiles, but possibly in long-term use in breeding new more potent varieties (Rodriguez-Morrison et al., 2021).

### 4.9 Minor cannabinoids

Minor cannabinoids are cannabionides that are produced in smaller quantities by the plant compared to THC and CBD. There are more than 120 different compounds that fall under this umbrella term, including cannabinol (CBN), cannabichromene (CBC), cannabigerol (CBG), cannabidiolonic acid (CBDa), cannabigerolic acid (CBGa), tetrahydrocannabinolic acid (THCa), cannabinolic acid (CBNa), cannabidivarin (CBDV), tetrahydrocannabivarin (THCV), cannabigerovarin (CBGV), cannabichromvarin (CBCV), and others (Hanuš et al., 2016; Gülck and Möller, 2020; Walsh et al., 2021).

In the experiment, UV radiation had no significant effect on the CBGa concentration in any group. The impact on CBGa might be limited due to the selective biosynthetic pathway activation, and the complex interaction between UV radiation and various plant factors. Also, no significant effect on the minor cannabinoid profile could be demonstrated for the graduated UV levels of the L3 groups, although the lateral CBN values were lower for all L3 groups than for the respective main shoots. Direct light exposure can increase the temperature of the flower. Higher temperatures can accelerate the enzymatic reactions that convert THCa to CBN, leading to higher CBN levels in these flowers. The UV treatment also had a significantly negative effect on the CBCa concentration in the L1 group compared to the control group L1_C. Cannabinoids can degrade when exposed to light, particularly UV light. CBCa may be more susceptible to photo-degradation than other cannabinoids, leading to a decrease in its concentration under UVB treatment.

### 4.10 Terpenes

In addition to cannabinoids, terpenes are increasingly becoming the economic focus of the cannabis industry. The aroma of the flower often determines the perception of the qualitative properties of the cannabis product (Gilbert and DiVerdi, 2018). These aroma characteristics are largely caused by terpenes.

Like cannabinoids, a positive influence of UV light on the terpene profile would be a welcome effect. Since a positive influence on the formation of terpenes has already been demonstrated in previous experiments with other plants, it is appears obvious that this is also potentially possible with *Cannabis sativa* L (Robson et al., 2019; Huché-Thélier et al., 2016).

Terpenes are hydrocarbons with small isoprene units. The number of isoprenes determines whether it is categorized as monoterpenes, sesquiterpenes, diterpenes and triterpenes (Sommano et al., 2020). In the experiment carried out, the desired positive effect of increased terpene content was only seen when treated with 1.8 w/m^2^ UV radiation (L3_1).

The idea of positively influencing the terpene profile with UV light is not new. In previous experiments with other plant species, for example, increased isoprene emission due to UV radiation was demonstrated. Isoprene is the basic unit of terpenes and in this case protected the leaves exposed to UV radiation from sudden heat (Tiiva et al., 2007; Liu et al., 2017).

For *Cannabis sativa*, a negative influence on the total terpene concentration was reported in previous experiments (Rodriguez-Morrison et al., 2021). However, the results of this study show that its different spectral distribution and intensity of the UV light allows an increase of the terpene concentrations. In particular, the terpene linalool was significantly influenced in this experiment. Linalool is a terpene commonly found in various plants, including cannabis, and is known for its floral and sweet aroma. Treatment with 1.8 w/m^2^ UV radiation (L3_1) led to a significant increase in the concentration of linalool in the cannabis flowers.

The other terpenes also showed a clear dependence on UV irradiation. However, at the two higher irradiation intensities L3_2 and L3_3, terpene concentrations were reduced in the mean value of the groups, but not significantly.

The increase in linalool concentration was an observation that deviates from the current study situation. In the current study on the influence of UV light on terpenes, Rodriguez found that myrcene and linalool concentrations decreased while caryophyllene and guaiol concentrations increased with increasing UV-PFD (Rodriguez-Morrison et al., 2021).

However, the comparability of the studies is questionable. In the leaf tissue of grapes (*Vitis vinifera* L.), it was observed that not only the intensity can have a significant effect on terpene production, but also the exposure time. Specifically, two groups were studied: one with low UVB irradiation (16 hours at 8.25 μW cm^-2^) and one with high UVB irradiation (4 hours at 33 μW cm^-2^). It was found that the low UV treatment increased the production of membrane-associated triterpenes, while the high UV exposure led to an increase in antioxidants and the sesquiterpenic stress hormone abscisic acid (ABA). These results illustrate that both the intensity and duration of UV exposure are decisive factors for the biochemical response in plants. For example, in the study described (Rodriguez-Morrison et al., 2021), plants were treated with higher concentrations for only 3.5 hours, whereas in the present study, lower concentrations were applied over the entire 12 hours of flowering illumination.

## 5 Conclusion

The great economic and medical benefits of secondary constituents of the cannabis plant form the basis for the great interest in the possibility of positively influencing valuable constituents such as cannabinoids and terpenes with external factors without reducing yield levels. The study carried out, showed predominantly negative effects for different UV compositions on both yield and flower quality. Only UVA radiation at the lowest intensity level resulted in a significant improvement in content for the terpene linalool. A closer look at the flower condition revealed a direct effect of UV radiation on the trichomes. This led to the consideration that the maximum of cannabinoids had already been exceeded at the time of harvest due to the UV radiation. Due to the many factors such as spectral quality, intensity, irradiation duration, irradiation time by day or cultivation stage, but also the genetics used, which can be adjusted during UV treatment, there are still several research approaches for further studies on the use of UV light in cannabis.

## Data Availability

The raw data supporting the conclusions of this article will be made available by the authors, without undue reservation.
